# Terahertz Spectroscopy of Amorphous WSe_2_ and MoSe_2_ Thin Films

**DOI:** 10.3390/ma11091613

**Published:** 2018-09-04

**Authors:** Gianpaolo Papari, Can Koral, Toby Hallam, Georg Stefan Duesberg, Antonello Andreone

**Affiliations:** 1 Dipartimento di Fisica, Università di Napoli “Federico II,” Piazzale Tecchio 80, I-80125 Naples, Italy; can.koral@fisica.unina.it (C.K.); andreone@unina.it (A.A.); 2 CNR-SPIN, UOS Napoli, via Cinthia, I-80126 Naples, Italy; 3 CRANN & AMBER Center, School of Chemistry, Trinity College Dublin, Dublin 2, Ireland; toby.hallam@ncl.ac.uk (T.H.); georg.duesberg@unibw.de (G.S.D.); 4 School of Engineering, Newcastle University, Merz Court, Newcastle upon Tyne NE1 7RU, UK; 5 Institute of Physics, EIT 2, Faculty of Electrical Engineering and Information Technology, Universität der Bundeswehr München, Werner-Heisenberg-Weg 39, EIT2, 85577 Neubiberg, Germany

**Keywords:** THz time domain spectroscopy, transition metal dichalcogenides, dielectric function, absorption, refractive index

## Abstract

Time domain spectroscopy is used to determine the THz electromagnetic response of amorphous transition metal dichalcogenides WSe_2_ and MoSe_2_ in thin-film form. The dielectric function is obtained using a rigorous transmission model to account for the large etalon effect. The Drude–Smith model is applied to retrieve the dielectric function, and from there, the sample conductivity.

## 1. Introduction

Transition metal dichalcogenides (TMDs) are characterized by a layered structure, responsible for their peculiar intrinsic 2-D electronic properties. Each crystal plane is based on the chemical configuration MX_2_, where M is a metal and X is a chalcogen [1,2]. Adjacent layers are held together by van der Waals forces, which inhibits free charge mobility across MX_2_ planes. TMDs in thin-film form offer a large variety of compounds with adjustable band gaps [3,4] and present a good transparency in the THz band that might pave the way to the development of a promising class of novel devices for THz optoelectronics [4]. Different from other tunable band gap systems like graphene, TMDs can also exploit an additional external parameter to control their own electro-optic properties as the magnetic field because of the spin-dependent band gap [5].

Furthermore, it has been shown [1,6,7] that the energy band structure of TMDs presents an interesting and unique dependence on the layer number, and a band gap transition from indirect to direct one for monolayer samples. Consequently, there is a consistent and growing demand in thin-film characterization, for the possible development of novel fast electronic [8,9] and/or electro-optical systems [10,11]. Recent advances in materials, sources, and detectors from the sub-THz and THz [3] to the mid-infrared [1,4,6] highlight the importance of time domain spectroscopy (TDS) to determine material properties at very high frequencies, such as the refractive index, absorption loss, and complex conductivity [12,13,14,15].

Nowadays, experimental strategies (see, for instance, Reference [16]), along with the application of proper theoretical models [17] and computational techniques [18,19,20,21], render the use of THz waves a robust and effective tool for thin-film characterization. Nevertheless, the presence in a majority of cases of a substrate unavoidably affects the fast (1–2 ps) time signal and produces a number of reflected copies, introducing sinusoidal (Fabry–Perot) oscillations in the Fourier transform of the transmitted and/or reflected wave. As long as the substrate is reasonably thick to induce a consistent time shift between the main signal and its copies, it is possible to cut them, applying a proper temporal windowing [22,23]. Of course, this procedure is recommended only when it does not affect the structure of the main signal. Otherwise, in the extraction of the electrodynamic parameters (EPs) of the sample under test, one runs the risk of losing relevant information.

Among several TMD composites, the Se based thin-films still lack investigation with linear THz spectroscopy. Here, we extract the electrodynamic parameters of amorphous WSe_2_ and MoSe_2_ films, 20 nm thick, deposited on a crystalline SiO_2_ substrate having a thickness of about 300 µm (*d* ≈ λ). Choosing a relatively thin substrate, the measurements suffer lower losses, but the transmitted signal shows a reflected copy too close to the main signal to be cut away. To minimize the unavoidable Fabry–Perot oscillations in EPs, we apply a reliable and simple method based on the transfer function (TF) equations [17]. In particular we retrieve the dielectric function ($\tilde{\varepsilon }$), and from there, we extract intrinsic parameters, such as the plasma frequency and the DC conductivity through the Drude–Smith model [24].

## 2. Thin-Film Fabrication and Characterization

### 2.1. TMD Deposition, and Morphological and Compositional Analysis

The TMD films have been deposited using a process known as thermally assisted conversion (TAC). TAC has the capability of depositing a continuous large-scale film with multiple layers [25]. The appropriate transition metal (Mo, W) films were deposited using a sputter coater (PECS, Gatan, Pleasanton, CA, USA) at <2 Å/s to form a smooth conformal coating 10 nm thick on a c-axis SiO_2_ substrate. In order to convert the metal films with their respective diselenides, they were exposed to selenium vapor at an elevated temperature in a home-built hot-wall chemical vapor deposition reactor with a process that has been described in more detail previously [3]. Specifically, metal films were converted to selenide in the reactor with chamber temperature at 600 °C under a flow of about 150 sccm of argon with the Se powder source held upstream at 219 °C for 2 h. Previous characterization indicated that the final thickness of selenide (MoSe_2_, WSe_2_) was 20 nm, which was double the initial metal thickness. To protect the films from oxidants in the atmosphere, they were encapsulated with 5 nm of Al_2_O_3_ using atomic layer deposition carried out over 46 cycles at 80 °C and a pressure of 2.2 Torr.

Using a shadow mask, samples were deposited on one half of SiO_2_ c-axis substrates having a total area of 2 cm × 1 cm. The second half was intentionally left bare, so that one could retrieve the thin-film parameters employing the exact film thickness and refractive index ${\tilde{n}}_{s}$ of the specific substrate on which each sample was deposited. As it will be shown in detail in the next paragraph, this was the only effective procedure to minimize the error introduced by the unavoidably slight difference in substrate parameters [16,23].

To evaluate the thickness profile and roughness, and check the morphology of the films under investigation, atomic force microscopy (AFM) measurements were carried out in a non-contact mode by using a Park Instruments XE-100 atomic force microscope in tapping mode.

To evaluate the chemical composition of the sample, X-ray photoelectron spectroscopy (XPS) was performed using a VG Scientific ESCAlab MKII system with a CLAM2 analyzer and PSP twin anode, unmonochromatized Al Kα X-ray source. Spectral fitting was performed using CasaXPS software. Charge compensation used the adventitious carbon peak at 284.6 eV. A Shirley background subtraction was performed, followed by the fitting of transition metal and chalcogen doublet peaks with mixed Gaussian and Lorentzian character.

### 2.2. Experimental Techniques

**Figure 1:**
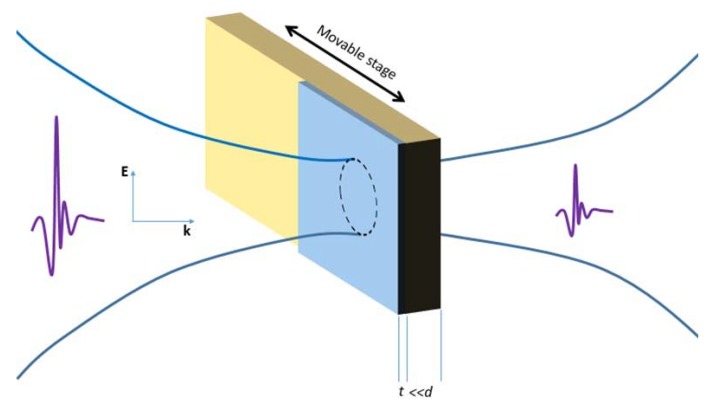
Sketch of the experimental setup. The blue area represents the film deposited on half the SiO_2_ substrate (depicted in yellow). A step motor controlled the position of the sample with respect to the impinging THz pulse.

The measurement setup was similar to the one adopted in other experiments performed earlier [26,27]. Briefly, the sample was mounted over a hollow support of aluminum allowing us to measure, in a row, the signal transmitted through the free space, $E_{air}\left(t\right)$, the bare substrate, $E_{sub}\left(t\right)$, and the film, $E_{film}(t$). A step motor controlled the position where the THz pulse impinged the support. The procedure is schematically shown in Figure 1. The size of the Gaussian profile of the focused THz pulse, estimated with the standard knife-edge technique, was of the order of 2 mm. In order to remove unwanted spectral features related to water absorption, measurements were performed in an N_2_ environment with a nominal humidity level below 1%. TDS was performed using a commercial THz system (Menlo Systems^TM^, Martinsried, Germany) that generated and detected a pulsed THz signal, having a repetition rate of about 80 MHz. The physical process, for both generation and detection, was based on the down conversion of an optical pulse exciting a photoconductive antenna nested over a GaAs film. Temporal pulsed signals, of duration 1–2 ps, were acquired for about 200 ps, resulting in a frequency resolution of about 5 GHz and a useful bandwidth in the interval 0.15–3 THz.

## 3. Theoretical Background

Routine procedures for the retrieval of the complex electrodynamic parameters of a single or a multilayer sample have been settled for years and goes under the name of the transfer function (TF) model [28,29]. Specifically, when the electromagnetic signal passes through a film having thickness *t* deposited on a dielectric substrate, the transfer function in the frequency domain can be written as [17]:

(1) $$\tilde{T}\left(\omega \right)={\tilde{E}}_{f}\left(\omega \right)/{\tilde{E}}_{s}\left(\omega \right)=2\frac{{\tilde{n}}_{f}\left({\tilde{n}}_{a}+{\tilde{n}}_{s}\right)}{\left({\tilde{n}}_{a}+{\tilde{n}}_{f}\right)\left({\tilde{n}}_{f}+{\tilde{n}}_{s}\right)}\exp\left\{-i\left({\tilde{n}}_{f}-{\tilde{n}}_{s}\right)\frac{\omega t}{c}\right\}\cdot FP\left(\omega \right)$$

where

$$FP\left(\omega \right)=\frac{1}{1-\left(\frac{{\tilde{n}}_{f}-{\tilde{n}}_{a}}{{\tilde{n}}_{f}+{\tilde{n}}_{a}}\right)\left(\frac{{\tilde{n}}_{f}-{\tilde{n}}_{s}}{{\tilde{n}}_{f}+{\tilde{n}}_{s}}\right)exp\left\{-2i{\tilde{n}}_{f}\omega t/c\right\}}$$

represents the Fabry–Perot factor [29]. Subscripts *a*, *s*, and *f* in the complex refractive index $\tilde{n}=n+ik$ refers to air, the substrate, and the TMD film respectively. Equation (1) suggests that ${\tilde{n}}_{f}$ and ${\tilde{n}}_{s}$ affect the modulus and phase of transmission in such a way that a specific numerical algorithm has to be applied [28]. Specifically, this algorithm, called the total variation technique (TVT) extracts the refractive index of the sample by minimizing the difference between the experimental and theoretical transmission curve [28]. We first extracted the refractive index ${\tilde{n}}_{s}$ of the substrate by employing the simple expression of the transfer function for a single layer [29]. The use of the TVT in the simple case of a thick substrate enabled us to get both ${\tilde{n}}_{s}$ and also the effective thickness *d_eff_* of the substrate [18]. In our measurements, ${\tilde{n}}_{s}$ as well as *d_eff_* values were extracted by using the commercial software Teralyzer^TM^ (Martinsried, Germany). Since *d_eff_* was determined with an accuracy of ±2 µm, this translates into a relative error lower than 1% for both the real and imaginary components of the substrate dielectric function. Once ${\tilde{n}}_{s}$ and *d_eff_* were known, Equation (1) was then applied to get ${\tilde{n}}_{f}=n_{f}+i\;n_{f}$ using the total variation technique routine, with a total relative error estimated to be about 10% for the samples under investigation. The error in the evaluation of ${\tilde{n}}_{f}$ was mostly related to the uncertainty in the film thickness due to the surface roughness that in our case was of the order of 4 nm. In the following graphs, this error was usually well within the scattered data point size.

In general, once $\tilde{n}$ is found, the real and imaginary parts of the dielectric function $\tilde{\varepsilon }$ could be obtained using the standard formulas $\varepsilon_{r}=n^{2}-k^{2}$ and $\varepsilon_{i}=2nk$.

Within the TF model, the effective thickness *d_eff_* and refractive index ${\tilde{n}}_{s}$ of the bare substrate are given by the values determining the minimum FP oscillations [19]. However, “residual ripples” are always present in all calculated electrodynamic parameters, hence a systematic approach to smooth them without altering the effective physical quantities is a recommended strategy. Towards this aim, it is worth first to mention that it is better to deal with FP residual oscillations only at the very end of the retrieval process, once a “universal” dielectric function (expressible as the sum of the monotonic trend and the oscillating contribution) $\tilde{\varepsilon }={\tilde{\varepsilon }}_{\mathrm{avg}}+{\tilde{\varepsilon }}_{\mathrm{osc}}$ has been determined with an accurate measure of the substrate thickness. In this way, a reference frequency interval ∆*f* to apply an adjacent point average procedure with is univocally determined by the physical condition on the extinction coefficient of the substrate: $k_{s}$(ω) ≥ 0. Then, the number of points used for the smoothing process is essentially set by the number of points contained in a single FP oscillation, which in our case was of the order of 50.

To describe the dependence of the complex dielectric/conductivity function on the intrinsic material parameters related to the mean dynamics of free charges, the standard and widely used approach is the Drude–Smith (DS) model [24]. In this framework the dielectric function $\tilde{\varepsilon }$ is given by

(2) $$\tilde{\varepsilon }\left(\omega \right)=\varepsilon_{\infty }-\left\{\frac{\omega_{p}^{2}}{\omega^{2}+i{\omega \omega }_{\tau }}\left(1+c_{1}\frac{\omega_{\tau }}{\omega_{\tau }-i\omega }\right)\right\}$$

where $\omega_{p}$ is the plasma frequency, $\omega_{\tau }$ = 1/τ is the relaxation frequency, $\varepsilon_{\infty }$ is an asymptotic constant, and $c_{1}$ accounts for the backscattered particles and assumes values between −1 and 0. From the dielectric function the complex conductivity can be easily obtained using the relation

(3) $$\tilde{\sigma }\left(\omega \right)=-i\left[\tilde{\varepsilon }\left(\omega \right)-\varepsilon_{\infty }\right]\varepsilon_{0}\omega$$

where $\varepsilon_{0}$ is the vacuum permittivity. After the fitting procedure, one can yield the sample zero frequency conductivity as $\sigma_{dc}=\left(1+c_{1}\right){\;\omega }_{p}^{2}\varepsilon_{0}/\omega_{\tau }$.

## 4. Results

**Figure 2:**
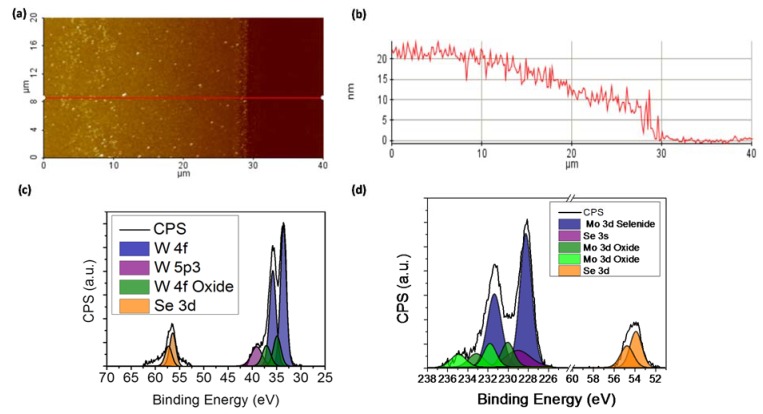
Characterization of TMD films. (**a**) AFM image of MoSe_2_. The area between 30 and 40 µm represents the bare SiO_2_ substrate. (**b**) Surface profile of MoSe_2_ according to the red line presented in panel (a). (**c**,**d**) XPS data and spectral fitting of WSe_2_ and MoSe_2_, respectively.

An AFM image of the MoSe_2_ sample is reported in Figure 2a. The image shows the step generated by the shadow mask between the substrate and the film enabling us to measure the surface profile along the red line as presented in Figure 2b. Because of the shadow mask, the MoSe_2_ thickness slowly decreased from about 20 nm to 0 with a roughness of 4 nm at most. The WSe_2_ sample was characterized by similar roughness, although the film edge was decorated by a series of flakes a few microns large

A core level XPS spectrum of WSe_2_ grown at 600 °C is shown in Figure 2c. A deconvolution process allowed for highlighting, through Gaussian-Lorentzian like curves, the binding energies of atoms composing the material. The majority of the metal was bound to selenium, which was manifest as the large doublet (blue) at 33 eV with some residual oxides present (green). The spin-orbit splitting in the W 4f doublets was 2.18 eV and the branching ratio (W 4f5/2 to W 4f7/2) was 3:4. A background contribution existed around the selenium peak (orange), which could have been due to excess amorphous Se on the surface. Using relative sensitivity factors (RSFs) and comparing the area under the peaks, the atomic ratio was found to be 1.7, with a possible underestimation due to the difficulty in deconvoluting the Se peak from the background.

As shown in Figure 2d, the Mo 3d core-level could be fitted with one main component at a binding energy of 228–229 eV for the Mo 3d5/2 peak (blue), which originated from selenised Mo, and one smaller component on the high binding energy side of the peak (green), which was most likely related to residual oxides [25]. The spin-orbit splitting in the Mo 3d doublets was 3.14 eV and the branching ratio (Mo 3d3/2 to Mo 3d5/2) was 2:3. All components of the Mo 3d line have been fitted using a mixed Gaussian-Lorentzian line shape with a FWHM of 1.3 ± 0.2 eV. These spectra were complicated to deconvolute by the fact that they lie on top of the Se 3s level, which has been fitted with a FWHM of 3.5 eV (purple) and normalized to the Se 3d. The Se 3d core-level (orange) could be fitted with only one component at a binding energy of 55 eV, indicating that there was no unreacted selenium on the surface. The stoichiometric atomic ratio was found to be close to 2. Thus, the oxide contributions were found to be minimal, below 5%, which was most likely a surface oxide, occurring while the samples were exposed to an ambient environment prior to XPS analysis.

**Figure 3:**
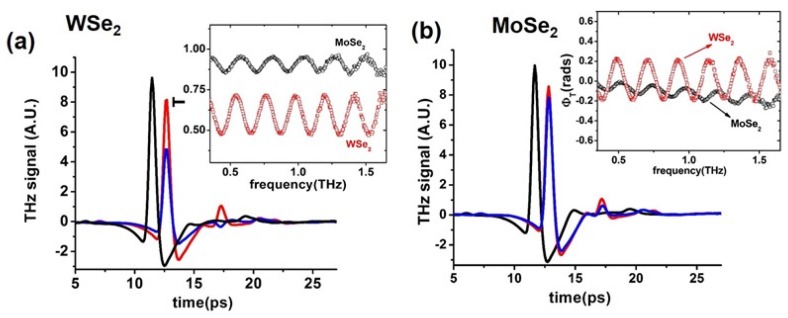
(**a**) Black, red, and blue solid lines represent the time dependent THz signals referred for free space, SiO_2_ substrate, and WSe_2_ film + substrate, respectively. (**b**) Similar to (**a**), with the blue curve showing the measurement on the MoSe_2_ film + substrate. Left and right insets: Comparison between the magnitude T(ω) and phase $\phi_{T}\left(\omega \right)$ respectively of the transmission function for the two TMD samples.

**Figure 4:**
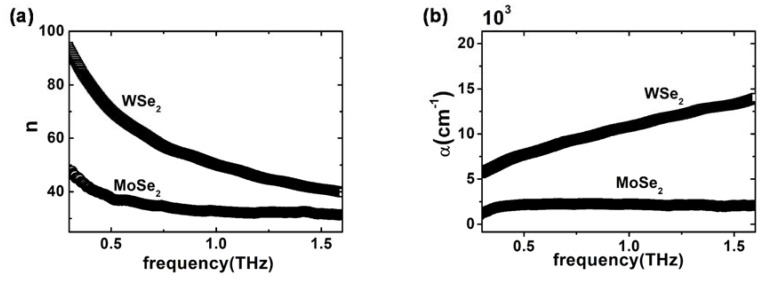

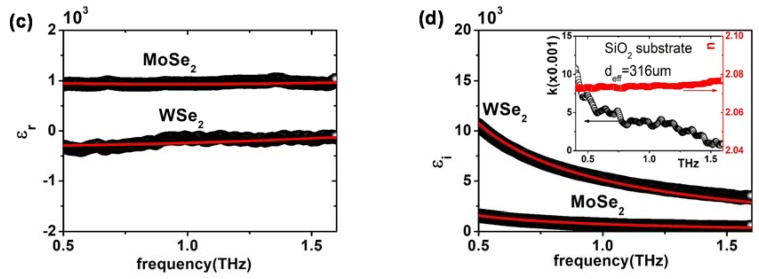
(**a**,**b**) show the refractive index *n* and the absorption coefficient respectively of the two samples. (**c**,**d**) report the real ($\varepsilon_{r}$) and imaginary ($\varepsilon_{i}$ ) parts of dielectric function of TMD’s films as extracted by using the TF model. The red line corresponds to the Drude–Smith model fit whose parameters are reported in Table 1. Inset: refractive index and extinction coefficient versus frequency of the SiO_2_ substrate.

In Figure 3a,b, the temporal evolution of the electric field signals transmitted across the WSe_2_ and MoSe_2_ samples, given by blue curves, is reported. In the same graphs, black curves represent the free space transmission, whereas red curves refer to signals passing through the bare substrate. Peak amplitude in the substrate was always around 20% lower than the free space case, and its delay of about δt ≈ 2 ps described the proper change in refractive index (since the nominal thickness was 300 µm, this corresponds to $n_{s}\;$≈ 2 for the crystalline SiO_2_). The presence of the WSe_2_ and MoSe_2_ thin-film induced a further reduction in the transmitted signal (blue curves) of about 30% and 10%, respectively, when compared to the bare substrate. In all cases, THz waves impinging and passing through each sample caused an etalon effect with the first reflected signal manifesting itself with a delay of almost 5 ps from the main peak. Time dependent signals were then converted in the frequency domain using a fast Fourier transform (FFT) method, since EPs are directly related to the transmission function $\tilde{T}\left(\omega \right)={\tilde{E}}_{f}\left(\omega \right)/{\tilde{E}}_{s}\left(\omega \right)$, where the complex quantities ${\tilde{E}}_{f}\left(\omega \right)$ and ${\tilde{E}}_{s}\left(\omega \right)$ are the FFT curves of the sample and bare substrate, respectively. In the inset of Figure 4a,b, the modulus and phase of the transmission functions for the two TMD samples are reported, respectively. Both quantities $\left|\tilde{T}\left(\omega \right)\right|$ and $\phi_{T}=Arg\left({\tilde{E}}_{s}\right)-Arg\left({\tilde{E}}_{r}\right)$ show a robust FP oscillation whose period was of the order of $\Delta f_{FP}\approx 0.2\;\mathrm{THz}$, in agreement with the fact that the first echo had a delay with respect to the main signal of the order of $\delta \tau \approx 1/\Delta f_{FP}\approx 2\delta t$ [29]. Since we observed no substantial difference between the THz signals acquired on a bare substrate and one covered by 5 nm of Al_2_O_3_, in the data extraction, we neglected the presence of alumina on the TMD surface. On average, the transmission modulus of the MoSe_2_ film was about 0.9, whereas in the WSe_2_ sample, it strongly oscillated around 0.6. As for the phase, in the case of WSe_2_, it remained substantially constant around zero, as opposed to the MoSe_2_, where it showed a slight decrease, reflected in a larger dynamics of refractive index [*n*(ω) ≈ 1 − *c* $\phi_{T}$(ω)/ω*d*] [29]. Applying the total variation technique, we found that the effective thickness of the substrates were *d_eff_* (WSe_2_) = (332 ± 2) μm and *d_eff_* (MoSe_2_) = (316 ± 2) μm. Using these values, we could then find ${\tilde{n}}_{s}=n_{s}+ik_{s}$. In the inset of Figure 4d, the refractive index and the extinction coefficient of the *c*-axis SiO_2_ substrate are reported. At *f* = 1 THz, $n_{s}$ = 2.05 ± 0.02 and $k_{s}\;$=$\left(4.00\pm 0.04\right)\times 10^{-3}$ in substantial agreement with previous works [30,31].

## 5. Discussion

The substrate refractive index can be used by Equation (1) to calculate ${\tilde{n}}_{f}$. In Figure 4a,b, the refractive index $n_{f}$ and the absorption coefficient $\alpha_{f}=2\omega k_{f}/c$ of the two TMD films are shown, respectively. WSe_2_ showed a higher value of both the refractive index and extinction coefficient compared to MoSe_2_. In particular, absorption in WSe_2_ was higher than in MoSe_2_ by almost a factor of 5, with a well-defined positive slope, whereas $\alpha_{f,\mathrm{MoSe}2}$ saturated at about 2000 cm^−1^ in the range of 0.5–1.5 THz. $n_{f}$ and $\alpha_{f}$ in the measured WSe_2_ and MoSe_2_ samples were consistent with those of a doped semiconducting material. Indeed, they were larger than GaAs [22], but lower than pure metallic thin-films [20].

Same results are presented in Figure 4c,d, here in terms of the real and imaginary part respectively of the complex dielectric function. The real part $\varepsilon_{r}$ (Figure 4c) displays substantial flat dynamics, with MoSe_2_ characterized by a very high dielectric constant (≈1000) and WSe_2_ values being slightly negative. Imaginary parts $\varepsilon_{i}$ of the two films are reported in Figure 4d, showing the expected free-charge decrease, although values for $\varepsilon_{i}$ (WSe_2_) were larger than for $\varepsilon_{i}$ (MoSe_2_) by a factor of 5 or more. The MoSe_2_ film, therefore, showed a semiconducting behavior [20,31] with the real part being strictly positive and a fast decay of $\varepsilon_{i}$ versus frequency [32]. On the other hand, the WSe_2_ sample clearly presented a weak metallic behavior in the THz region, showing a complete negative $\varepsilon_{r}$ and large values for $\varepsilon_{i}$.

Fitting curves on the retrieved dielectric functions of WSe_2_ and MoSe_2_ films, obtained via the DS model, are reported as continuous red lines in the same figures. The used fitting parameters are reported in Table 1, with an uncertainty of about 5%.

The higher conductivity of WSe_2_ with respect to MoSe_2_ mainly resulted in larger values of $\omega_{p}$ and smaller values of $c_{1}$, as expected.

Nevertheless, the frequency response of the dielectric function and the values retrieved for the DC conductivity using the DS model was in clear contrast to what is observed in single crystal samples [33]. The observed metallic behavior may be ascribed to the presence of conductive channels in grain boundaries. A high density of metallic grain boundaries can in fact spontaneously form in slightly Se-deficient Mo thin films grown by molecular-beam epitaxy molecular-beam epitaxy [34]. We speculate that the same mechanism may apply to WSe_2_ samples. This would also explain the larger values of conductivity in the latter case.

## 6. Conclusions

We have retrieved the dielectric function of conducting MoSe_2_ and WSe_2_ TMDs by using time domain terahertz spectroscopy. Thin films have been created via a thermally assisted conversion process, which produced samples with good homogeneity. XPS measurements revealed the presence of a residual portion of uncombined metal particles, and therefore a TM:Se ratio smaller than 1:2. Ab initio thermodynamic simulations have shown [35] that this may favor the formation of dichalcogen-deficient grain boundaries instead of Se vacancies during the growth process. We have extracted the electrodynamic parameters of thin films deposited on 300 µm thick substrates, which introduce a relatively strong FP effect in transmission data. By applying a rigorous protocol based on the TF model and a self-consistent moving average procedure, we have minimized the periodic FP oscillations and fitted $\tilde{\varepsilon }$ with the Drude–Smith function. The relatively high conductivity observed in both compounds may be ascribed to a high density of line defects presenting a metallic character.

The relatively high transparence along with the conducting features are encouraging in view of a possible use of TMD-based thin-films for applications as tunable metadevices in the THz region. The observed conducting nature can represent a promising alternative for the realization of new devices with higher currents and lower contact resistance.

## Figures and Tables

**Table 1:** Best fit parameters for the dielectric functions of WSe_2_ and MoSe_2_ films using the DS model. Parameter accuracy is of the order of 5%.

Sample	$\omega_{p}\;\left(THz\right)$	$\omega_{\tau }\;\left(THz\right)$	$c_{1}$	$\sigma_{dc\;}\left(S/cm\right)$	$\varepsilon_{\infty }$
WSe_2_	850	16	−0.22	3000	1200
MoSe_2_	270	9.5	−0.35	444	1220
