# Correction: G. Papari et al. Terahertz Spectroscopy of Amorphous WSe_2_ and MoSe_2_ Thin Films. *Materials* 2018, *11*, 1613

**DOI:** 10.3390/ma13041017

**Published:** 2020-02-24

**Authors:** Gian Paolo Papari, Can Koral, Toby Hallam, Georg Stefan Duesberg, Antonello Andreone

**Affiliations:** 1 Dipartimento di Fisica, Università di Napoli “Federico II,” Piazzale Tecchio 80, I-80125 Naples, Italy; can.koral@fisica.unina.it (C.K.); andreone@unina.it (A.A.); 2 CNR-SPIN, UOS Napoli, via Cinthia, I-80126 Naples, Italy; 3 CRANN & AMBER Center, School of Chemistry, Trinity College Dublin, Dublin 2, Ireland; toby.hallam@ncl.ac.uk (T.H.); georg.duesberg@unibw.de (G.S.D.); 4 School of Engineering, Newcastle University, Merz Court, Newcastle upon Tyne NE1 7RU, UK; 5 Institute of Physics, EIT 2, Faculty of Electrical Engineering and Information Technology, Universität der Bundeswehr München, Werner-Heisenberg-Weg 39, EIT2, 85577 Neubiberg, Germany

The exponential factor in Equation (1) of the paper published in *Materials* [1] reports a misprint and the correct expression of the transmission function is

(1) $$\tilde{T}\left(\omega \right)=\frac{{\tilde{E}}_{f}\left(\omega \right)}{{\tilde{E}}_{s}\left(\omega \right)}=2\frac{{\tilde{n}}_{f}\left({\tilde{n}}_{a}+{\tilde{n}}_{s}\right)}{\left({\tilde{n}}_{f}+{\tilde{n}}_{a}\right)\left({\tilde{n}}_{f}+{\tilde{n}}_{s}\right)}exp\left\{-\;i\;\left({\tilde{n}}_{f}-{\tilde{n}}_{a}\right)\frac{\omega t}{c}\right\}FP\left(\omega \right)$$

where

$$FP\left(\omega \right)=\frac{1}{1-\left(\frac{{\tilde{n}}_{f}-{\tilde{n}}_{a}}{{\tilde{n}}_{f}+{\tilde{n}}_{a}}\right)\left(\frac{{\tilde{n}}_{f}-{\tilde{n}}_{s}}{{\tilde{n}}_{f}+{\tilde{n}}_{s}}\right)exp\left\{-i\;2\;{\tilde{n}}_{f}\frac{\omega t}{c}\right\}}$$

These changes have no material impact on the conclusions of the paper.

The authors would like to apologize for any inconvenience caused to the readers by these changes.

## References

[1] Papari G., Koral C., Hallam T., Duesberg G.S., Andreone A. (2018). Terahertz Spectroscopy of Amorphous WSe_2_ and MoSe_2_ Thin Films. Materials.

